# Could drought conditions trigger Schmallenberg virus and other arboviruses circulation?

**DOI:** 10.1186/1476-072X-12-7

**Published:** 2013-02-14

**Authors:** Mattia Calzolari, Alessandro Albieri

**Affiliations:** 1 Laboratorio Entomologia Sanitaria, Istituto Zooprofilattico Sperimentale della Lombardia e dell’Emilia Romagna “B. Ubertini”, Via Pitagora 2, Reggio Emilia, 42100, Italy; 2 Centro Agricoltura Ambiente “G. Nicoli”, Via Argini Nord 3351, Crevalcore, 40014, Italy

**Keywords:** Drought, Arbovirus, Standardized precipitation index, Schmallenberg virus, Usutu virus, West Nile virus

## Abstract

**Background:**

In 2011, a new orthobunyavirus, named the Schmallenberg virus (SBV), was discovered in Europe. Like the related Shamonda virus, SBV is an arbovirus (arthropod-borne virus). After its discovery, the virus was detected in a wide area in north-western Europe, an unexpected finding in a territory where climatic conditions would not seem ideal for arbovirus transmission. This sudden expansion suggests the effect of 2011 drought as a key factor that may have triggered SBV circulation. The possible influence of drought, recorded in north-western Europe in early 2011, on virus circulation was evaluated.

**Methods and results:**

The locations of SBV detections in Europe until April 2012 were obtained, and area of virus circulation was evaluated by kernel density estimation. Precipitation data in SBV circulation area, summarized by the 3 month precipitation indexes of May, were compared with precipitation data outside that area, confirming driest conditions in that area.

**Conclusions:**

The onset of drought conditions recorded in the SBV detection area in early 2011 may have promoted the circulation of this virus. A correlation between circulation of some arboviruses and drought has been reported elsewhere. This was mainly explained by an effect of water deficit on the environment, which altered the relationships between vectors and reservoirs, but this correlation might be also the result of unknown effects of drought on the vectors. The effect of drought conditions on arbovirus circulation is most likely underestimated and should be considered, since it could promote expansion of arboviruses into new areas in a global warming scenario.

## Background

The Schmallenberg virus (SBV) causes congenital malformations and stillbirths in cattle, sheep, and goats. Clinical signs of infection in adult animals are either absent or non-specific, but fever, loss of appetite, reduction in milk yield, and diarrhoea have been recorded in cattle [1,2]. Therefore, even if this virus does not represent a risk for humans [3], it could result in significant economic losses in farming [4].

The expansion of SBV in north-western Europe was the second event of spread of an arbovirus in this area, after the unexpected arrival of Blue tongue virus 8 (BTV-8) in 2006 [5]. While these two viruses are not related (BTV belongs to Reoviridae family), both share the same vectors: culicoids (*Culicoides* spp.), which are biting midges of the Ceratopogonidae family [2,4]. The surveillance efforts performed in northern European countries to monitor BTV-8 did not previously detect the SBV. Together with the available epidemiological data, this allows the onset of SBV circulation to be estimated as May 2011 [2]. The geographical spread of SBV over this short period was impressive, and much faster than that of BTV-8; it was probably promoted by the absence of immunity in the local population of domestic ruminants. Several factors may have influenced the SBV circulation, but one key factor, which occurred in 2011, stands out as possibly enabling this expansion: drought.

In Italy, an enhancing influence of drought was reported in mosquito circulation of the Usutu virus (USUV) [6], a flavivirus belonging to the Japanese encephalitis complex. The first detection of this virus in Europe dates back to 2001 in Austria [7], but it now seems to be endemic in several European countries [8]. This virus is transmitted by mosquitoes, mainly of the *Culex* genus, and has its main reservoirs in wild birds [9], although the possible involvement of other vertebrates in its cycle has been hypothesized [6].

The influence of drought conditions was previously reported for the circulation of other two flaviviruses that, similarly to Usutu, are maintained by a bird-mosquito transmission cycle: St. Louis encephalitis virus (SLEV) [10,11] and West Nile virus [10,12-14] (WNV). In 2012, drought conditions were also recorded in the Balkans and northern Italy, where a large number of human cases of WNV disease were reported [15], and several counties in the United States were affected by both drought and a WNV outbreak, with a large increase of human cases [16]. While the medical importance of USUV is not fully understood [8,17], risks of WNV and SLEV for human health are well recognized. In Africa, an association with unusually dry, warm conditions was recorded for the circulation of chikungunya virus (CHIKV) [18,19], a mosquito-transmitted alphavirus. Human beings could be reservoirs for CHIKV, which represents a major health problem in the world [20].

The epidemiology of culicoid-borne viruses is not well known as the mosquito-borne viruses one, because of the gaps in knowledge of biting midge taxonomy and biology [21], nevertheless an enhancing effect of drought was suggested on epizootic hemorrhagic disease (EHD), caused by a virus (EHDV) closely related to BTV, in United States cervids [22]. Moreover, severe drought conditions did not prevent outbreaks of congenital defects in domestic ruminants in Australia, caused by Akabane virus [23], which is closely related to SBV [4].

Interestingly, in 2011, an event of drought, characterized by the greatest deficit in accumulated rainfall in May, was recorded in north-western Europe, particularly in areas in which SBV was subsequently reported [24]. The possible influence of this event on SBV circulation was tested by comparing precipitation data in the estimated area of SBV circulation with precipitation data outside that area.

## Results and discussion

**Figure 1:**
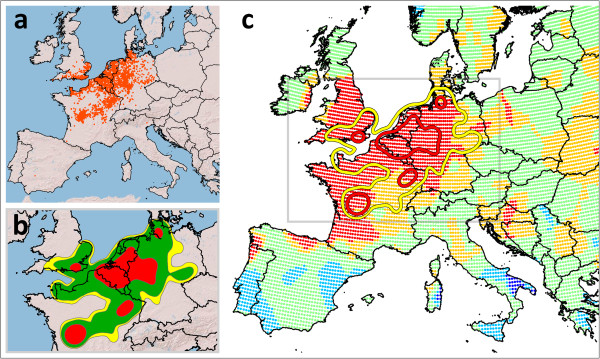
**Drought conditions in Schmallenberg virus (SBV) circulation area in 2011.** The sites of SBV detection until April 2012 are shown on Europe map (**a**). These sites were utilized to evaluate the area of virus circulation by kernel density estimation (KDE) (**b**). Then (**c**) 95% and 50% volume contours of KDE (yellow line and red line respectively) were overlaid to the map showing the interpolated 3 month standardized precipitation indices (SPI3) of May 2011. SPI3 value: red <-2, orange >-2 <-1, green >-1 <1, azure >1 <2 , blue>2. SPI3 indices were provided by European Drought Observatory [24], due to the improvement in measurements, future SPI values, referring to the same period, may be slightly different.

A dataset of 3,309 SBV positive sites was obtained (Figure 1a), with an average distance of 6,381 meters between sites (minimum distance 1 m, maximum distance 628,774 m). The average nearest neighbour analysis (ANNA) confirmed the clustering of SBV locations (p<0.01, Z-Score= - 66.47). The kernel density estimation (KDE) produced a single SBV circulation area, diffused from Germany to England and France (Figure 1b).

The average of the 3 month standardized precipitation indices (SPI3s) inside the obtained 95% KDE volume contour was -2.7 (±0.8 s.d., *n* 1,069) and the average outside of the contour was -0.2 (±1.0 s.d., *n* 22,951) (graphically represented in Figure 1c, yellow line). This means that more intense drought conditions occurred in the estimated SBV circulation area during the quarter from March to May 2011, with rainfalls that deviated by -2.7 units from the seasonal average, while outside of this area, a deviation of only -0.2 units was recorded.

A more restrictive contour was obtained by the 50% of the KDE, which produced four areas of more intensive circulation (Figure 1b, red). The largest of these areas – between Belgium, Germany and the Netherlands – includes the site in which the virus was discovered and the area of the first recording of the related disease symptoms [1,2,4], indicating that it was probably the initial area affected by the virus. Interestingly, the average of the SPI3 indexes in that area was -3.4 (±0.4 s.d., *n* 208) (graphically represented in Figure 1c, red line), lower than that of the 95% KDE contour. The occurrence of drought conditions inside the SBV circulation area, in the same three-month period, was confirmed by the low rainfall and largest number of consecutive dry days respect to the surrounding area (Table 1). Additionally, the extent of the average and maximum temperature was higher inside the SBV circulation area (Table 1). Indeed, the lack of rain affects the environmental moisture, which affects the temperature, so it is difficult to discern, during drought conditions, the specific effects of these factors on virus circulation.

**Table 1:** Evaluation of differences in weather conditions recorded by climatic stations in/out the SBV circulation area

	**Average stations in SBV area (s.d.)**	**Average stations out SBV area (s.d.)**	***p-*value**
Total rainfall (mm)	64.8 (16.9)	164.1 (96.3)	<0.01
Consecutive dry days	21.2 (6.4)	19.4 (8.2)	<0.01
Maximum temperature (°C)	18.0 (2.1)	17.4 (3.8)	<0.05
Average temperature (°C)	11.6 (1.6)	11.1(3.8)	<0.01
Minimum temperature (°C)	5.3 (1.5)	5.1 (3.0)	>0.05
Temperature range (°C)	12.7 (2.2)	12.3 (2.7)	<0.05

Moreover, the possible influence of livestock density on the SBV distribution was evaluated, although the low-density areas seem to slow down the spread of the virus, a strict correlation between the animal density and SBV diffusion was not found (Additional file 1).

These results strongly suggested that the drought conditions recorded in early 2011 had an enhancing effect on the spread and circulation of the SBV.

The apparent effect seems counterintuitive: drought conditions generally have negative effects on the vector density [9,11], and a decrease in virus circulation would logically be expected. However, this expected response does not constantly occur and a high density of vectors does not invariably mean higher viral circulation [4]; other factors come into play, such as host preferences of vectors, the density, availability and immunity level of the reservoirs, and may strongly influence the arboviral load in the environment.

The hypothesis made to support the action of drought on the arbovirus circulation is mainly based on the decrease in food resources and suitable habitats caused by drought. This decrease could have forced vectors and vertebrate hosts into limited areas, thereby increasing the possibility of vector-host contact and viral circulation. In previous studies, the reported influence of drought on WNV and SLEV circulation was explained by a spatial congregation of vectors and hosts in limited suitable areas, which promoted viral circulation between reservoirs and vectors [11,13], or by an effect of decreasing abundance of mosquito predators and competitors [25]. The hypothesis presented for CHIKV, which does not require an enzootic amplification to produce epidemics, was that drought increased the importance of domestic water storage in vector breeding, with a subsequently more probable vector-human contact [19]. Alternatively, a habitat change could have increased the density of larval breeding sites of the mosquito vectors [18]. However, these ecological explanations do not match the observations for the SBV, as the congregation of livestock that could act as reservoirs of this virus was not affected by drought, since the water sources for livestock are artificial, therefore not strictly rainfall-dependent. In addition, the breeding sites for culicoids are not as dependent on water as are those of mosquitoes.

Consequently, other mechanisms, not yet investigated, could exist, and a direct effect of drought on the vectorial competence of the insects could be hypothesized, as has been proposed for the widely described action of temperature on the virus susceptibility of mosquitoes [26,27] and culicoids [28,29]. Adverse conditions, like drought, could affect mosquito bionomics, as demonstrated by the decrease in egg production in the *Culex pipiens* mosquito following repeated bouts of dehydration [30]. In a similar way, drought could facilitate infection of the insects or influence the extrinsic incubation period length, a factor important in the vector capacity. Alternatively, the infected insects may have an unknown advantage during drought conditions over those not infected.

## Conclusions

To the best of our knowledge, a possible direct effect of drought on arbovirus vectors has never been specifically investigated, and further studies are needed to confirm the extent of this phenomenon. However, the obtained results and literature indications highlight a possible drought association with viral circulation, and on the ability of drought to potentially enhance the circulation of arboviruses in a territory. Therefore, drought conditions could act very significantly on the arbovirus economy, and might have played a crucial role in the recent emergence of some of these viruses.

If this effect is confirmed, and given the probable increase in drought events in the near future [31] due to global warming, an unexpected and marked expansion of some arboviruses may occur in areas previously considered to be immune to this problem.

## Methods

The European precipitation data from February to May, 2011, summarized by the SPI3, interpolated to a decimal degree quarter grid, were provided by the European Drought Observatory. The SPI3 is a statistical indicator of drought and compares the total precipitation received at a particular location during 3 months with the long-term rainfall distribution for the same period of time at that location. The accumulated precipitation is standardized to allow the statistical comparison of wetter and drier climates, and the SPI results are given in units of standard deviation from the long-term mean of the standardized distribution [24].

Data from European meteorological stations, in the interval 43°N–57°N of latitude and 11°W–20°E of longitude, were downloaded from site of European Climate Assessment & Dataset project (ECA&D) [32]. The average of daily temperature (minimum, maximum, average), the average of temperature range, the maximum number of consecutive dry days and the precipitation sum in the three-mount period, from March to May 2011, for available stations were obtained. The differences between average of indices from stations inside and outside the SBV circulation area (95% volume contour area of SBV KDE) were evaluated by *t* test.

The 2011 total number of bovine animals, sheep and goats were obtained for European territorial units (NUTS) available in the Agriculture database of statistical office of the European Union (Eurostat) [33]. Livestock density was obtained for every NUTS dividing the total number of animals by the entire NUTS area.

The locations of SBV detections were obtained from the EMPRES Global Animal Disease Information System [34]. The SBV activity in the 2012 season was excluded by considering only cases reported until April 2012. Data that were lacking from Belgium [35] were added to this database. The pattern of spatial data was characterized by conducting ANNA on the SBV locations.

KDE was performed using ESRI ArcGIS 9.3 and the Spatial Analyst extension and was applied to this dataset to evaluate the area of virus circulation (Figure 1b). The KDE is a geospatial technique based on the kernel function (a bivariate probability density function) used to create a surface to indicate the intensity of the events of a phenomenon [36]. A bandwidth size of 100 km was chosen, considering the ability of culicoids to spread the virus this distance [2,5]. The area of SBV circulation was estimated by the 95% and 50% volume contour of the SBV KDE, which represents the boundary of the area that contains 95% and 50% of the volume of the obtained KDE (Figure 1b, yellow).

## Abbreviations

SBV: Schmallenberg virus; BTV: Blue tongue virus; USUV: Usutu virus; SLEV: St. Louis encephalitis virus; WNV: West Nile virus; CHIKV: Chikungunya virus; ANNA: Average nearest neighbor analysis; KDE: Kernel density estimation; SPI3: 3 month standardized precipitation index

## Competing interests

The authors declare that they have no competing interests.

## Authors’ contributions

MC performed the analysis and drafted the manuscript. AA analysed data in geographic information system. Both authors read and approved the final manuscript.

## Supplementary Material

Additional file 1 **Livestock density and Schmallenberg virus (SBV) circulation area in 2011.** The 2011 livestock densities (bovine animals, sheep, goats), obtained for the different European territorial units, are shown with the overlaying of the 95% and 50% volume contours of KDE of SBV (yellow line and red line respectively). Click here for file
