# Supplementary material for: Could drought conditions trigger Schmallenberg virus and other arboviruses circulation?
Source: Int J Health Geogr. 2013 Feb 14;12:7. doi: 10.1186/1476-072X-12-7 (PMC3614475; doi:10.1186/1476-072X-12-7)

Livestock density and Schmallenberg virus (SBV) circulation area in 2011. The 2011 livestock densities (bovine animals, sheep, goats), obtained for the different European territorial units, are shown with the overlaying of the 95% and 50% volume contours of KDE of SBV (yellow line and red line respectively).

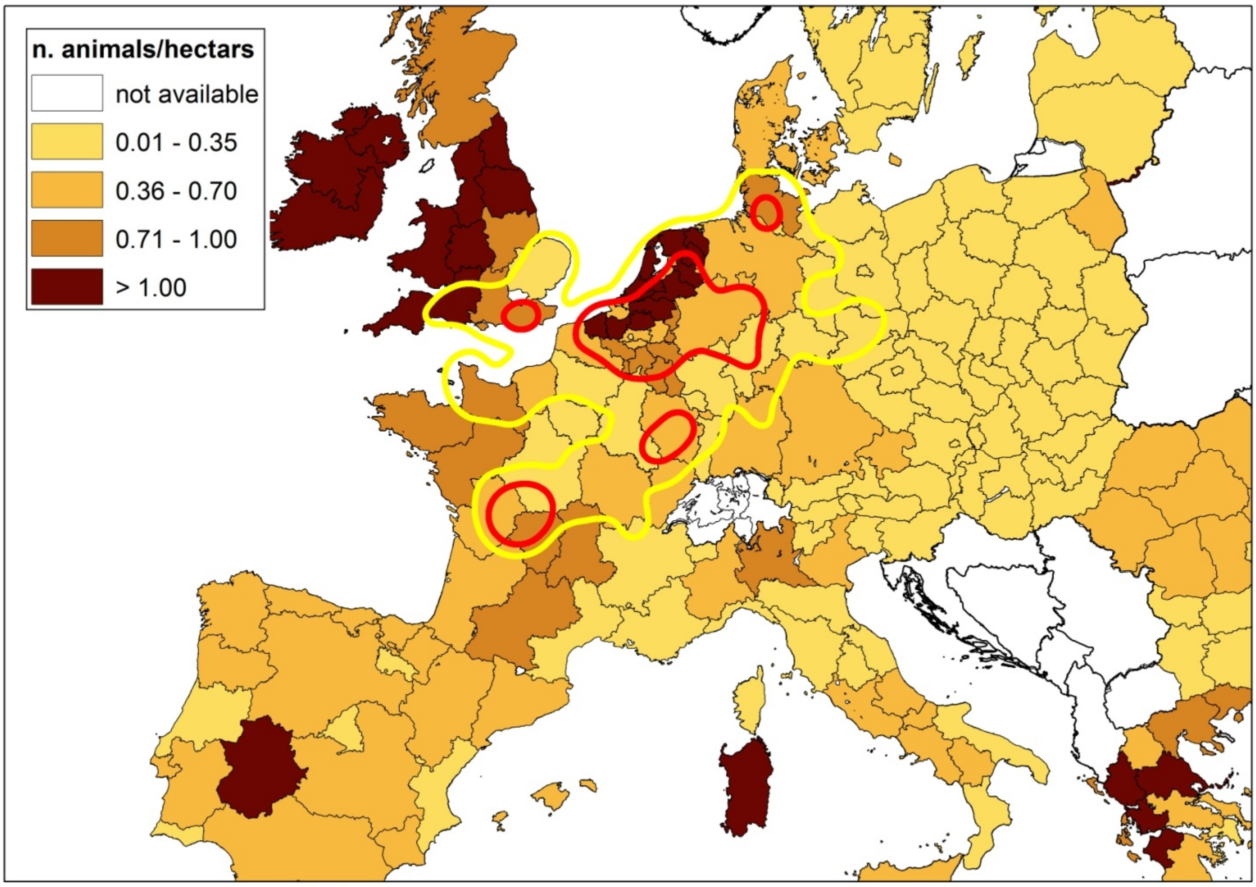

Supplement: Additional file 1 — Livestock density and Schmallenberg virus (SBV) circulation area in 2011. The 2011 livestock densities (bovine animals, sheep, goats), obtained for the different European territorial units, are shown with the overlaying of the 95% and 50% volume contours of KDE of SBV (yellow line and red line respectively). [file 1476-072X-12-7-S1.pdf]
